# Comparison of Three Fixation Methods for Femoral Neck Fracture in Young Adults: Experimental and Numerical Investigations

**DOI:** 10.1007/s40846-015-0085-9

**Published:** 2015-10-01

**Authors:** Shabnam Samsami, Sadegh Saberi, Sanambar Sadighi, Gholamreza Rouhi

**Affiliations:** Faculty of Biomedical Engineering, Amirkabir University of Technology, 424 Hafez Ave, Tehran, 15875-4413 Iran; Department of Orthopaedy, Tehran University of Medical Sciences, Tehran, 14155-6447 Iran; Iranian Tissue Bank and Research Center, Tehran University of Medical Sciences, Tehran, 14158-868 Iran

**Keywords:** Vertical femoral neck fracture, Fracture fixation, Static loading, Cyclic loading, Bone fracture healing, Stability, Interfragmentary movement, Finite element analysis

## Abstract

Femoral neck fractures in young patients are usually caused by a high-energy trauma, which results in a perpendicular fracture. Although efforts are focused on preserving the femoral head in young patients, vertical femoral neck fracture is a problematic orthopedic injury due to the domination of shear forces. Due to controversy regarding which fixation method is the best choice, the purpose of this study was to find the most stable fixation method for this kind of fracture. This study includes experimental testing on cadaveric bone samples and finite element analysis (FEA) for three fracture fixation techniques, namely cannulated screws (CSs), dynamic hip screw with derotational screw (DHS + DS), and proximal femoral locking plate (PFLP). Experimental results of bone-implant stiffness, average femoral head displacement, failure load, failure energy, and relative position of the fractured fragments indicate that DHS + DS offers the strongest structure for stabilizing a vertical femoral neck fracture. Experimental data and FEA results both indicate that under static loading, the DHS + DS method of fixation produces the lowest femoral head displacement and interfragmentary movement, followed by PFLP and then CSs. The results of this research suggest that, based on the clinical assumption that a restricted weight-bearing regimen is recommended in the postoperative rehabilitation protocol, the DHS + DS method of fixation is a better choice compared to CSs and PFLP for a vertical femoral neck fracture fixation in young adults.

## Introduction

Femoral neck fracture in young patients is usually due to a high-energy trauma, which results in a vertical fracture. A common injury pattern in this population is a vertical shear fracture. Because of the domination of shear forces, vertical femoral neck fracture is a problematic orthopedic injury. In young adults, preservation of a femoral head that requires stable fixation is vital [1–3].

In general, bone healing is divided into direct and indirect bone healing. Indirect or secondary bone healing consists of the sequential steps of tissue differentiation, bone resorption, and uniting of the fracture fragments by external callus. Finally, the fracture undergoes long-lasting internal remodeling [4]. Direct or primary bone healing skips the intermediate steps of tissue differentiation and bone resorption and progresses directly to the final internal remodeling of the Haversian system [5]. Primary bone healing that follows stable fixation and compression can be divided into gap healing and contact healing, both of which are able to achieve bone union without external callus formation and any fibrous tissue or cartilage formation within the fracture gap [4]. Previous studies demonstrated that in secondary bone healing, dynamized fixation and controlled axial micro-movement better stimulate callus formation and cortical bone healing in comparison to rigid fixation [6, 7]. Moreover, a reduction of load transfer by delaying full weight bearing is advantageous for the healing of fractures stabilized with flexible fixation systems [8]. Unlike secondary bone healing, direct bone healing requires rigid stabilization that suppresses the formation of a callus in either cancellous or cortical bone. Since most fractures are treated in a way that results in some degree of motion, primary healing is rare [4]. The biological aspects of damage to the blood supply, necrosis, and temporary porosity explain the importance of avoiding extensive contact of the implant with bone [9]. Locked plates and conventional plates rely on different mechanical principles to provide fracture fixation and different biological environments for healing. Locked plates may increasingly be used for indirect fracture reduction, while conventional plates may continue to be used for periarticular fractures, which demand perfect anatomical reduction [10].

It is noteworthy that for several reasons, the union of a femoral neck fracture should be of the primary type of bone healing process, which necessitates absolute stability at the fracture site [5]. First, the nature of the fracture (intracapsular) makes the fracture more vulnerable to non-union. Also, synovial fluid prevents blood clot formation, thereby eliminating an important factor, which contributes to secondary bone healing [5]. In addition, the intracapsular part of the femoral neck has no periosteal layer to participate in the bone healing process, so this kind of fracture can heal by endosteal union alone. Hence, the goal of internal fixation of an intracapsular femoral neck fracture is stable fixation with compression over the fracture fragments. If stable fixation is achieved, then a direct bone healing process can be expected for this kind of fracture [5].

According to a recent clinical study [3], despite timely, excellent reduction and accurate implant placement, the nonunion rate was 19 % for vertical femoral neck fractures treated with cannulated screws (CSs) alone, and 8 % for those treated with a fixed-angle device. Although these failure rates are not significantly different, because of the challenging nature of this fracture pattern, the ideal fixation device remains an open question [3]. To date, only a few biomechanical studies have evaluated the fixation stability of vertically oriented fractures of the femoral neck (Pauwels’ III femoral neck fracture) [11–18]. Several internal fixation methods have been used for the treatment of vertical femoral neck fracture with various clinical and biomechanical results [3, 11–14, 16–18]. A recent retrospective clinical study confirmed better union rates for vertical femoral neck fractures treated with fixed-angle devices compared with those for CSs alone [3]. Earlier biomechanical studies demonstrated that a dynamic hip screw (DHS) with a derotational screw (DS), DHS + DS, is superior to parallel CSs [19]. Locking plate technology, which allows multiple points of fixed-angle fixation into short epiphyseal segments, has recently been investigated for the fixation of this kind of fracture [12]. It was reported that the Synthes proximal femoral locking plate (PFLP) (Synthes, Paoli, PA, USA) provides the strongest fixation of fresh-frozen cadaveric samples with vertical femoral neck fractures compared to those provided by multiple parallel CSs and conventional fixed-angle implants. Although the intertan nail (IT) possesses some biomechanical benefits for the internal fixation of unstable femoral neck fractures compared with DHS and CSs, clinical studies are required to confirm the use of the IT as the ideal fixation method for unstable fractures of the femoral neck [16, 18]. To sum up, the results of recent biomechanical studies showed that the construct stiffness of fixed-angle devices is superior to that of CSs alone for the fixation of a Pauwels’ III femoral neck fracture [11–14].

Previous studies of femoral neck fractures have measured the instability of the fracture after fixation through the apparent increase in the fracture gap after osteotomy and reduction [19]. The aim of the present study was to compare biomechanical stability and bone healing feasibility for three fracture fixation techniques, namely CSs, DHS + DS, and PFLP. A series of experimental tests was performed on cadavers and finite element (FE) models were developed. The experimental techniques used in this study applied motion capture analysis to evaluate the relative motions between the fractured fragments. Thus, the stability of fixed fracture, a prerequisite of primary bone healing, was investigated [20].

## Materials and Methods

This study includes experimental and numerical sections that compare three common fixation methods for vertical formal neck fractures. In the experimental section, the stability of cadaveric bone samples fixed with various techniques is compared by considering biomechanical parameters during loading, namely stiffness, femoral head displacement, failure load, failure energy, and relative positions of fractured fragments. In the finite element analysis (FEA) section, static loading was simulated and the effect of fixation method on the mechanical performance of the proximal femur model was evaluated in terms of the femoral head displacement and interfragmentary movement. Finally, the results of experimental section were used to validate the FE models.

### Mechanical Tests

Three fresh-frozen cadaveric femurs from male donors with no known previous history of hip pathology were harvested at autopsy. The average age of donors was 47.7 ± 1.15 years. The donors had died in accidents or of acute disease without known long periods of immobilization. The bone mineral density and the intact stiffness of each specimen were measured by dual-energy X-ray absorptiometry (DEXA) and mechanical testing, respectively. The average values of the bone mineral density and stiffness were 0.57 ± 0.04 g/cm^2^ and 1.10 ± 0.01 N/mm, respectively. In order to preserve the mechanical properties of harvested samples, the specimens were cleaned of soft tissue and stored at -20 °C. All samples were thawed at room temperature for 6 h before testing, and sprayed intermittently with normal saline to keep them hydrated.

**Fig. 1:**
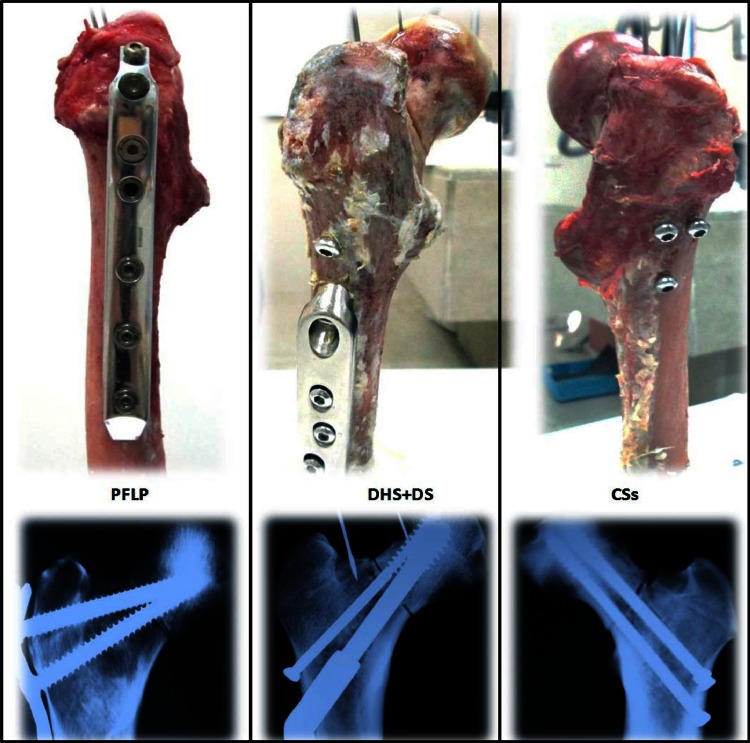
Fractured femurs fixed using PFLP, DHS + DS, and CSs

**Fig. 2:**
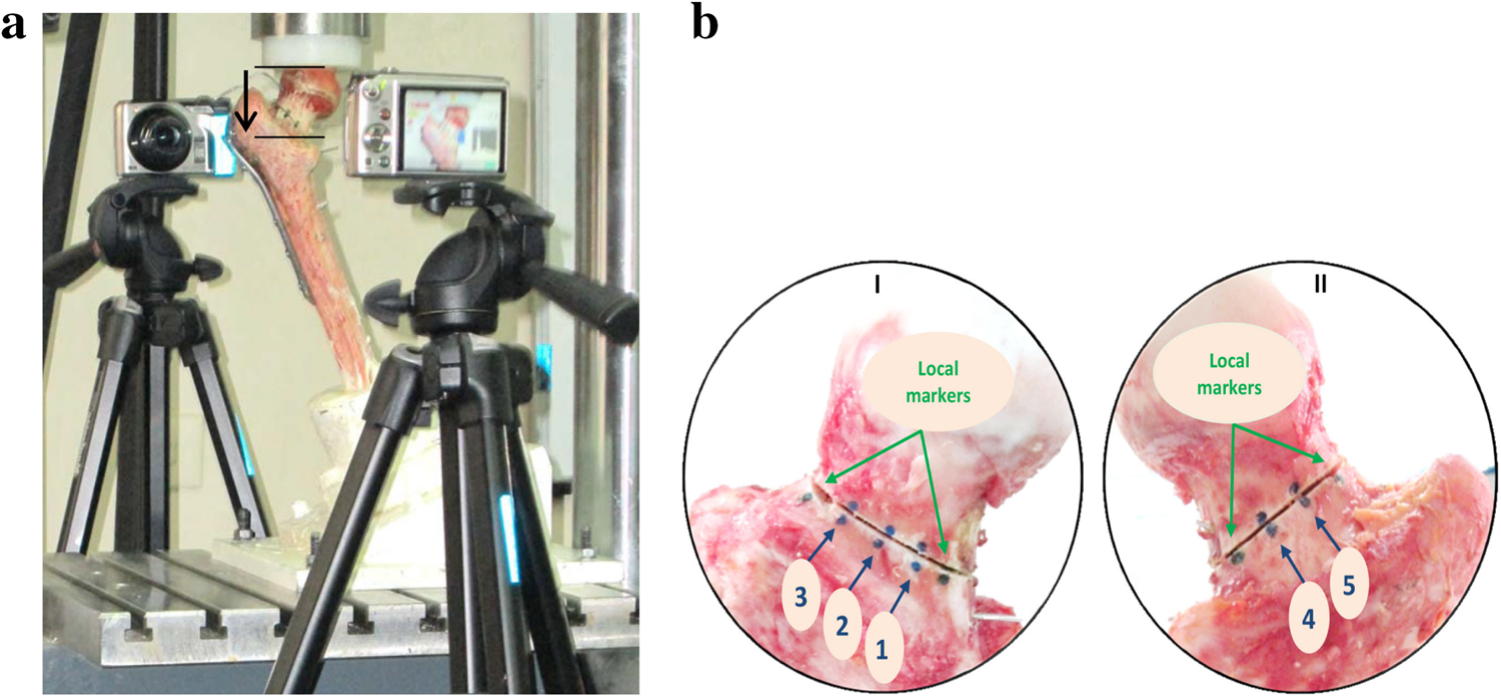
**a** Test setup (*black arrow* shows axial femoral head displacement). **b** Femoral neck viewed from (I) anterior side and (II) posterior side, showing osteotomy with visible markers. Locations of markers are (*1*) ant-inf, (*2*) ant-mid, (*3*) ant-sup, (*4*) pos-inf, and (*5*) post-sup

Vertical fractures (Pauwels’ III fracture, i.e., at 70° to the horizontal) were artificially produced in cadaveric proximal femurs by an orthopaedic surgeon, and fixed using various implants (i.e., CSs, PFLP, and DHS + DS) (Fig. 1). Then, all three samples were positioned at 25° of adduction, and loaded using a quasi-acetabulum fixture in incremental, cyclic, and failure phases (Fig. 2) [21]. In the CSs sample, three 7.3-mm stainless steel CSs (thread length: 32 mm) were inserted into the femoral head in an inverted triangle configuration, parallel to the femoral neck axis. The most inferior screw was positioned in the calcar region, above the lesser trochanter. The two cephalad screws were inserted superiorly, 5 mm from the anterior and posterior cortices of the femoral neck, and 5 mm from subchondral bone [12]. In the DHS + DS sample, a 135°, 3-hole DHS plate (made of stainless steel) was positioned with the central screw directed into the middle of the femoral head. The tip of the screw was seated 5–10 mm from subchondral bone. Three 4.5-mm cortical screws were used to fix the side plate to the femoral shaft. A superior neck 7.3-mm cannulated cancellous derotational lag screw was inserted parallel to the central screw [14]. Finally, in the PFLP sample, a fixed-angle PFLP (made of stainless steel) was secured with two locking screws in the femoral head: one 7.3-mm cannulated conical screw at 95° to the plate shaft, and one 5.0-mm cannulated conical screw at 110° to the plate shaft. The ends of all two screws were positioned 5 mm from subchondral bone. The side plate was fixed to the proximal femur using four 4.5-mm non-locking screws. All implants used in this study were made by Pooyandegan Pezeshki Pardis (3P) Company (Iran).

In order to measure interfragmentary movement, five pairs of markers (three on the anterior surface and two on the posterior side) were placed around the osteotomy, 10 mm apart from each other, as well as one marker on the femoral shaft, and the corresponding marker on the adjacent femoral head (Fig. 2) [20]. During the loading phases, the relative movement of each pair of markers was traced by a digital Casio EX-FH100 camcorder (10.1-megapixel high-speed digital camera). Two camcorders positioned at a distance of 35 cm from the sample and perpendicular to the plane of movement were used to measure two-dimensional (2D) movements of fracture fragments in the posterior and anterior aspects of the human femur. Before loading, for each aspect, a calibration frame that included a piece of graph paper was placed on the plane of motion, and the camcorder was focused on the markers and zoomed in until the calibration object occupied 1280 pixels × 720 pixels. As a result, each pixel equaled 0.1 mm in distance. The relative positions of fractured fragments were traced during loading using HD movie recording (30 fps). For 2D motion capture analysis of fracture fragments, 160 frames were extracted from the recorded movies for cyclic loading. Moreover, the initial movie of the static loading phase was converted to one with a frame rate of 1 fps. Then, the extracted frames were converted to the appropriate movies and imported into SkillSpector V.1.3.2 (Video4-coach, Denmark). The loading steps used to simulate partial weight-bearing in the immediate postoperative period were as follows [12, 22–24]: (I) incremental loading: each specimen was loaded to a maximum of 700 N at a rate of 1 mm/min displacement before and after fixation; (II) cyclic loading: each fixed sample was tested under sinusoidal cyclic loading, in which a 100–700 N force was applied at a frequency of 3 Hz for 10,000 cycles (this number of cycles approximates the expected interval for fracture consolidation) [12, 22]; and (III) failure loading: survived specimens were loaded at a rate of 1 mm/min until the failure criterion, defined as axial femoral head displacement or fracture displacement of equal or greater than 5 mm or instability in the load–displacement curve [22]. It is worth mentioning that the failure criterion used in this study was based on a fracture displacement of 5 mm or more. Moreover, the failure energy was calculated as the area under the force–displacement curve of the failure loading phase, from a displacement of zero up to the defined failure point.

Intrafragmentary motion was quantified based on Eq. (1) to transform the pixel locations into relative interfragmentary motion to determine the movement of the fracture gap. In this equation, *RM* is the femoral head motion relative to the shaft; *X*_*H*_ and *Z*_*s*_ are the locations of the markers on the head and shaft, respectively, in the X direction; *Y*_*s*_ and *Y*_*H*_ are the locations of the markers on the shaft and head, respectively, in the Y direction [20].

1 $$RM = \sqrt {(X_{H} - X_{S} )^{2} + (Y_{H} - Y_{S} )^{2} }$$

Considering the orientation of the fracture plane relative to the global X axis (*θ*), it was possible to transfer the marker locations to the local coordinate system using Eqs. (2)–(4), where (*x*_*L*_,*y*_*L*_) is the local coordinate position of a marker, *θ* is the orientation of the fracture plane with respect to the global x axis, (*X*_*1*_,*Y*_*1*_) is the position of the lower marker on the fracture plane, (*X*_*2*_,*Y*_*2*_) is the position of the upper marker on the fracture plane, and (*X*_*G*_,*Y*_*G*_) is the global coordinate position of the markers. Thus, changes in the relative position of each pair of markers in the x direction of the local coordinate system, parallel to the fracture line, show shear movement of fractured fragments. Also, changes in the relative position of each pair of markers in the y direction of the local coordinate system (perpendicular to the fracture line) correspond to axial motion of the fractured fragments [20].

2 $$\theta = \tan^{ - 1} ((Y_{2} - Y_{1} )/(X_{2} - X_{1} ))$$

3 $$x_{L} = (X_{G} - X_{1} )\cos \theta - (Y_{G} - Y_{1} )\sin \theta$$

4 $$y_{L} = (X_{G} - X_{1} )\sin \theta + (Y_{G} - Y_{1} )\cos \theta$$

In each loading step, the maximum interfragmentary movement of each pair of markers was compared among the three fixation methods.

### Finite Element Analysis

**Fig. 3:**
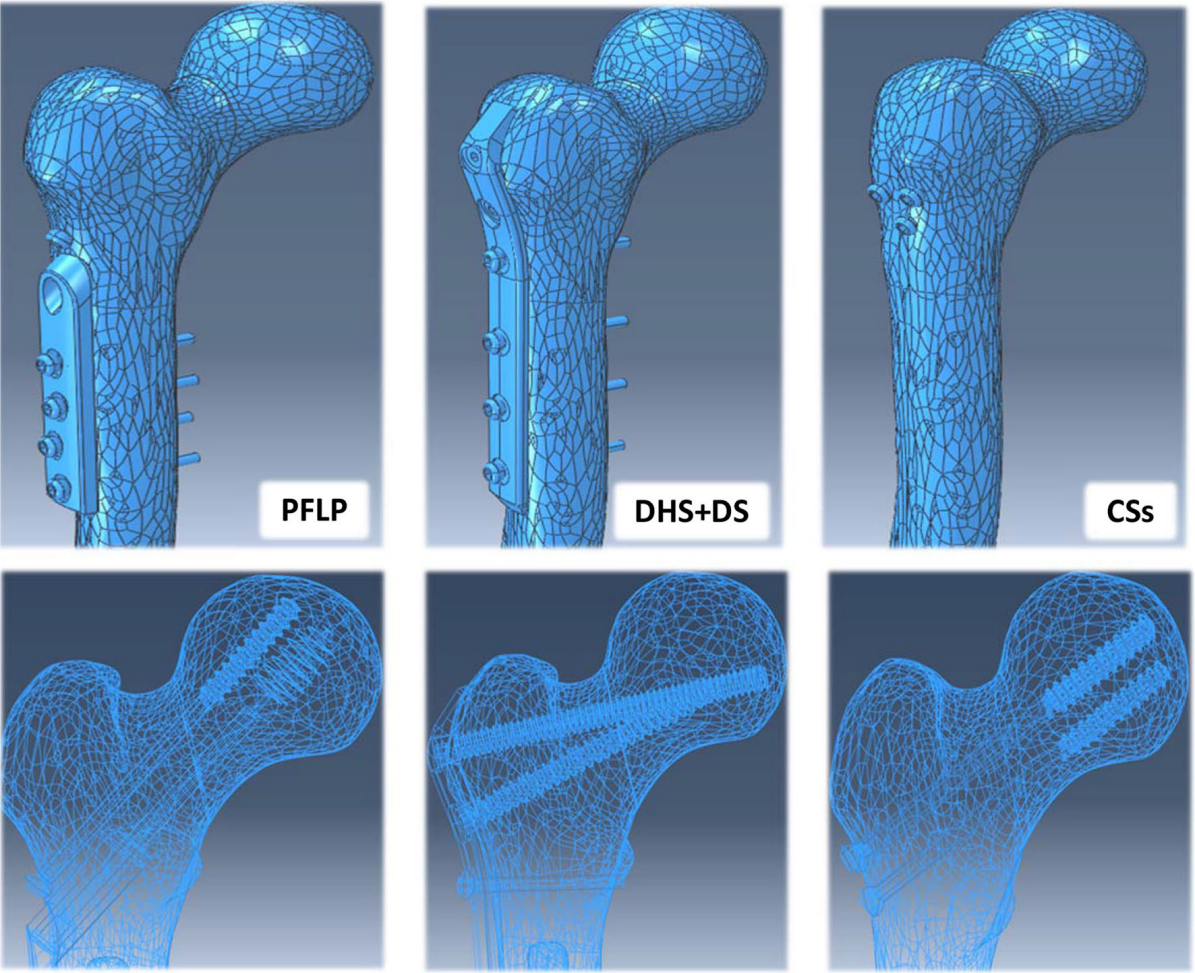
Geometric models of three fixation methods prepared for FEA

In order to compare the mechanical behavior of the fixation methods (CSs, DHS + DS, and PFLP), their FE models were generated. The geometric model of the proximal femur was developed from one set of CT images of a healthy man aged 65 years (image resolution = 512 × 512 pixels, pixel size = 0.33 mm, slice thickness = 1.25 mm, slice increment = 1.25 mm). CT images were imported into Mimics V.10.01 (Materialise NV, Belgium) and Catia V.5R.21 (Dassault Systèmes, France) to make three-dimensional (3D) geometry of the proximal femur. The geometric models of the implants were obtained using a coordinate measuring machine and Solid Works 2011 (Dassault Systèmes). The cortex screw threads were replaced by a smooth surface for simplicity, the size of which corresponds to the mean diameter of the thread [25, 26]. In order to prepare the geometric models, the intact bone models were segmented, the fractures were reduced, and the implants were positioned (Fig. 3).

**Fig. 4:**
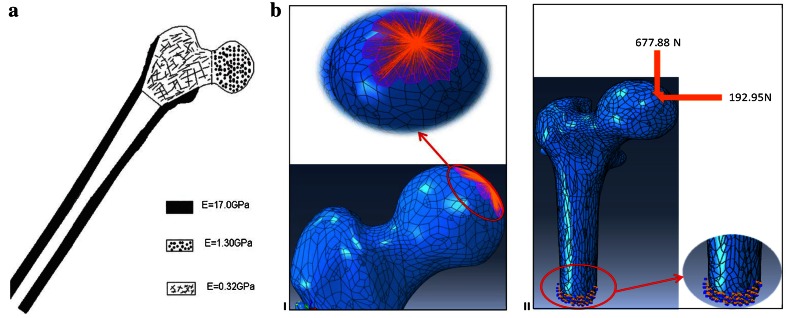
**a** Distribution of cortical and cancellous bone in proximal femur model: *a* cortical bone, *b* low-density cancellous, and *c* high-density cancellous bone [28]. **b** (I) Illustration of distributed coupling used for hip contact force. (II) Fully fixed boundary conditions in distal end of proximal femoral model and loads applied to model

For FEA, all geometric models were imported into ABAQUS V.6.10 (Dassault Systèmes). Stainless steel, the main material constituent of all three implants, was modeled as a homogeneous isotropic and elastic material, with an elastic modulus of E = 200 GPa and a Poisson’s ratio of μ = 0.3 [26, 27]. In all models, the bone tissue was assumed to be homogeneous, isotropic, and elastic. The elastic moduli of the cortical as well as high- and low-density cancellous bones were chosen to be 17.0, 1.3, and 0.32 GPa, respectively (Fig. 4) [28]. Even though it is well known that bone is an anisotropic and non-homogeneous material, since the focus of this study is to compare the performance of three fracture fixation methods, the choice of isotropic material properties for the bone is acceptable for modeling the human femoral bone. A Poisson’s ratio of 0.3 was assumed for the bone [28]. According to Fongsamootr et al. [29], the friction coefficient between bone and bone can be assumed to be 0.3. Thus, in all FE models, the space between individual fragments of the fractured femur was modeled using normal contact of the “HARD” type, with a friction coefficient of f = 0.3 [25, 30]. The same method was also used for modeling the contact between the DHS plate and the bone (f = 0.3) in the DHS + DS model [25, 31]. The screw-bone interface in all cases was assumed to be fixed, i.e., the tie contact condition was used, in order to reduce the computational time and increase the stability of numerical analyses [25, 31–33]. The purpose of the FE analysis was to simulate the static phase of the mechanical tests for the three fixation methods. Therefore, the distal ends of the proximal femur models were fully fixed. To apply the external force, a distributed coupling was used, by which single forces acting in a control reference node were equally distributed to the bone tissue at contact points of the femoral head with the acetabulum [25]. During the analyses, the models were loaded using the horizontal and vertical components of the hip contact force corresponding to the one leg stance position with a partial weight bearing assumption (see Fig. 4).

For each model, a free mesh using tetrahedral 10-node elements was computed using the ABAQUS mesher. The second-order shape functions of these elements ensured a mesh that was close enough to the bone’s boundary surfaces. Hexahedral elements are known to be more accurate than tetrahedral ones, but the complexity of our model did not enable us to use them [29]. Therefore, the individual parts of the broken femur and fixation implants were created using volume second-order tetrahedral C3D10 elements. The results were converged to the parameter of interest, i.e., the axial femoral head displacement, with about 152,000 and 211,000 elements depending on the fixation methods.

### Measurements

In the experimental section, stiffness, relative stiffness (i.e., the ratio of the stiffness of the bone-implant composite structure after fixation to the stiffness of unfractured bone), axial femoral head displacement, failure load, failure energy, and interfragmentary movement were measured in order to compare the stability, shear resistance, and feasibility of bone healing among the three fixation methods.

In FEA, Eq. (5) was used to calculate the average value of relative motion of fracture fragments.

5 $$Relative \, motion_{ave} = \sqrt {copen_{ave}^{2} + cslip1_{ave}^{2} + cslip2_{ave}^{2} }$$

where *copen*_*ave*_, *cslip1*_*ave*_, and *cslip2*_*ave*_ are the average separation of the fracture fragments and the average femoral head sliding relative to the femoral shaft in tangential directions, respectively. Because of the comparative purpose of this study, in both experimental and numerical sections, the FEM results (axial femoral head displacement and interfragmentary movement) of each model were divided by the corresponding results for the DHS + DS model, and the obtained values are reported as normalized results. For example, the FEM results of interfragmentary movement for DHS + DS and CSs are 0.017 mm and 0.034 mm, respectively, so the normalized interfragmentary movement for CSs model is 2 (shown in Fig. 10).

## Results

### Mechanical Tests

The stiffness, relative stiffness, failure load, and failure energy for the DHS + DS method of fixation were about 54, 78, 236 and 706 % higher than those for PFLP, respectively, and the axial femoral head displacement of this method was 43 % lower than that for PFLP. Moreover, the biomechanical parameters of the DHS + DS method (stiffness, relative stiffness, failure load, and failure energy) were about 66, 105, 320 and 515 % higher than those for CSs, respectively, and the axial femoral head displacement of this technique was 55 % lower than that for CSs (see Table 1).

**Table 1:** Relative stiffness, axial femoral head displacement, failure load, and failure energy of CSs, DHS + DS, and PFLP

Fixation method	Stiffness (N/mm)	Relative stiffness	Axial femoral head displacement (mm)	Failure load (kN)	Failure energy (J)
DHS + DS	404.3	0.41	2.58	5.67	13.46
PFLP	262.8	0.23	4.52	1.68	1.67
CSs	243.1	0.20	5.78	1.35	2.19

**Fig. 5:**
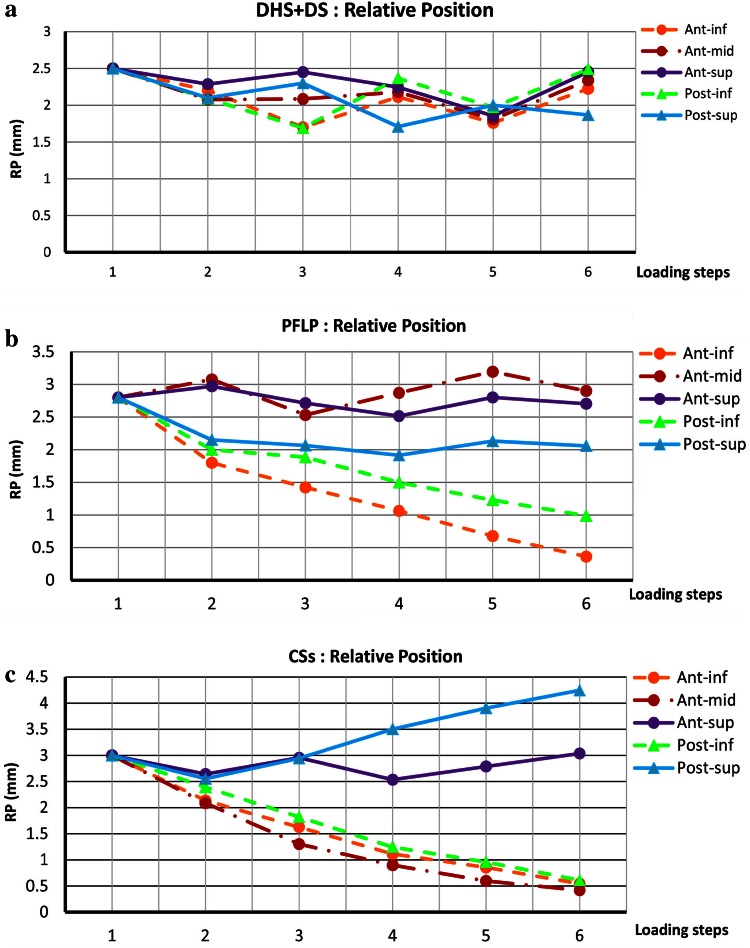
Relative position of fractured fragments (RP = √((X_s_ − X_H_)^2^ + (Y_s_ − Y_H)_^2^) versus various loading steps for various locations around fracture site. Step 1: initial position; step 2: incremental loading (at maximum load); steps 3–6: cyclic loading. **a** DHS + DS, **b** PFLP, and **c** CSs

Figure 5 shows the maximum change in the relative position of the fractured fragments at each loading phase for the tested samples. The interfragmentary movement curves of the DHS + DS sample show oscillatory trends for all locations around the fracture site. In contrast, for the CSs and PFLP samples, the trends of interfragmentary movement versus load were dissimilar for different locations around the fracture site. Moreover, small differences exist between the curves of post-inf and post-sup locations, and oscillatory trends for ant-mid and ant-sup locations represent less change in the relative position of the fractured fragments for the PFLP sample compared to that for the CSs specimen.

**Fig. 6:**
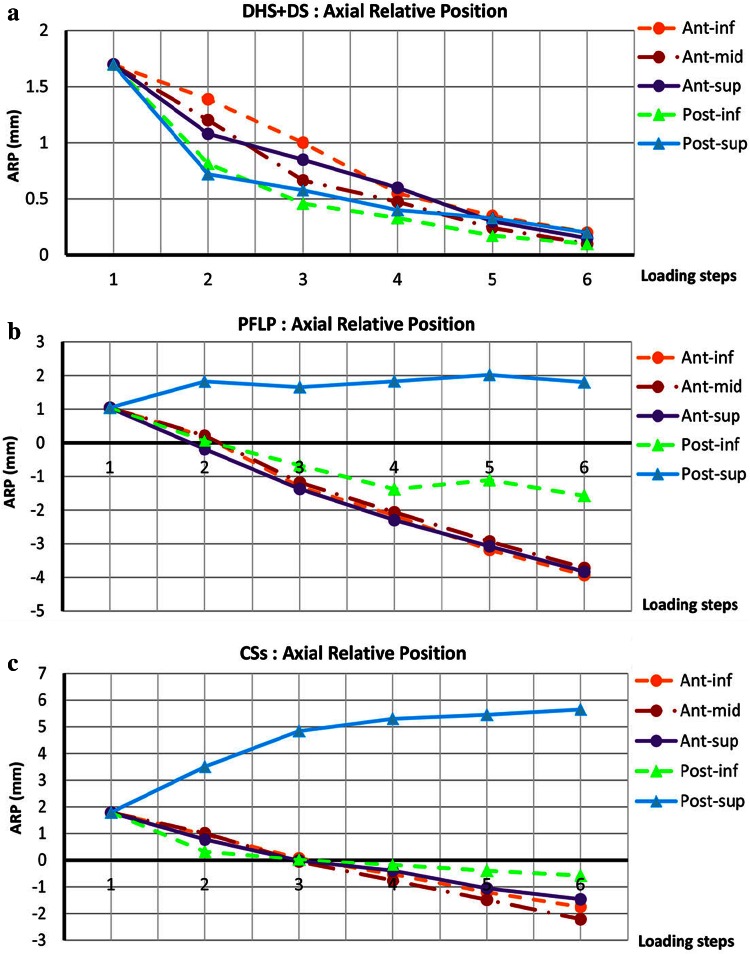
Axial relative position of fractured fragments (ARP = y_s_ − y_H_) versus various loading steps for various locations around fracture site. Step 1: initial position; step 2: incremental loading (at maximum load); steps 3–6: cyclic loading. **a** DHS + DS, **b** PFLP, and **c** CSs

Figure 6 shows the maximum change in the axial relative position of fractured fragments, i.e., $ARP = y_{S} - y_{H}$, at each loading phase for the tested specimens. Axial interfragmentary movement versus load curves of the DHS + DS sample show descending trends for all locations around the fracture site. For the PFLP specimen, the axial interfragmentary motion-load curves display descending trends and a change in the sign from positive to negative at ant-inf, ant-mid, and ant-sup locations. Moreover, at the post-inf- location, the axial interfragmentary movement decreases during loading, but this curve exhibits fewer gradients compared to those in other descending curves. Also, at the post-sup- location, the axial interfragmentary movement-load curve shows an oscillatory trend. For the CSs sample, the axial interfragmentary motion-load curves exhibit descending trends and a change in the sign from positive to negative at ant-inf, ant-mid, ant-sup, and post-inf locations. Moreover, at the post-sup- location, the axial relative position of fractured fragments increases during loading.

**Fig. 7:**
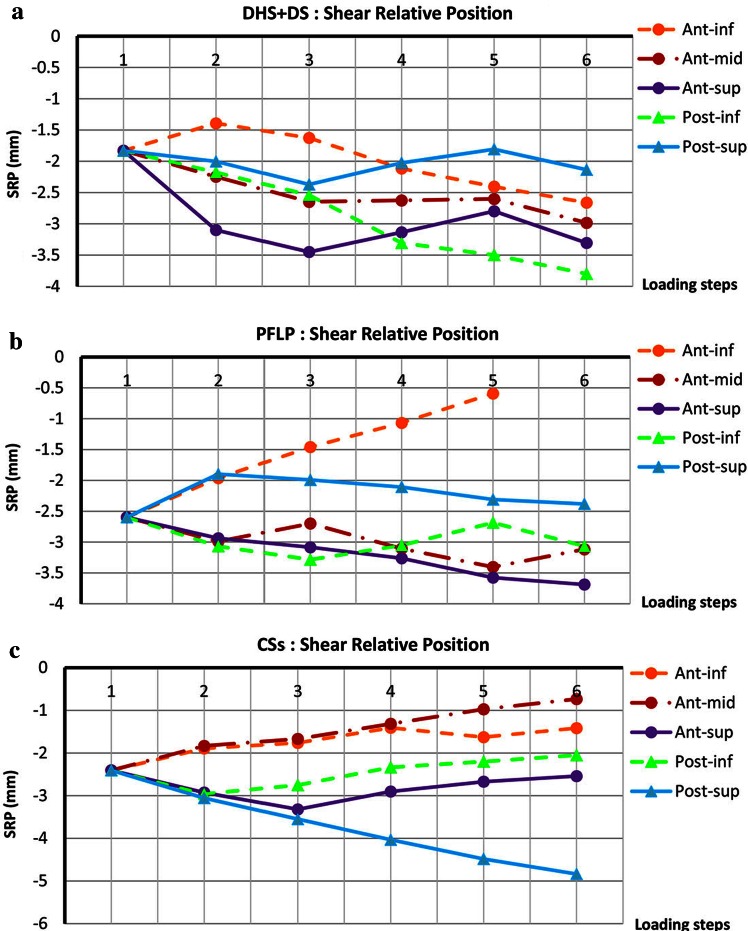
Shear relative position of fractured fragments (SRP = x_s_ − x_H_) versus various loading steps for various locations around fracture site. Step 1: initial position; step 2: incremental loading (at maximum load); steps 3–6: cyclic loading. **a** DHS + DS, **b** PFLP, and **c** CSs

Figure 7 shows the maximum change in the shear relative position of fractured fragments, i.e., $SRP = x_{S} - x_{H}$, at each loading phase for the tested specimens. The shear interfragmentary movement curves of the DHS + DS sample display oscillatory trends at an-mid, ant-sup, and post-sup locations. Also, the post-inf and ant-inf curves indicate that the shear interfragmentary movement decreases during loading. For the PFLP sample, the shear interfragmentary movement versus load curves show oscillatory trends at ant-mid and post-inf locations, and exhibit descending trends at post-sup and ant-sup locations. Moreover, at the ant-inf location, the shear interfragmentary movement increases during loading. For the CSs sample, the shear interfargmentary movement-load curves display ascending trends at ant-inf, ant-mid, ant-sup, and post-inf locations. Also, at the post-sup- location, the shear interfragmentary movement decreases during loading.

**Fig. 8:**
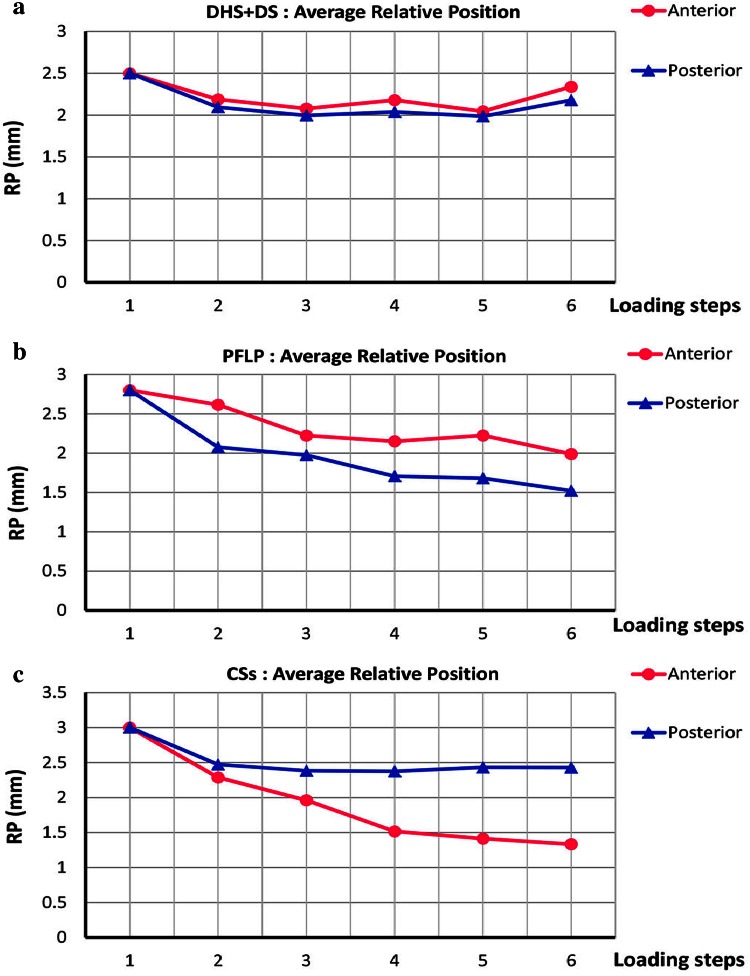
Average relative position of fractured fragments in anterior and posterior aspects versus various loading steps for **a** DHS + DS, **b** PFLP, and **c** CSs (step 1: initial position; step 2: incremental loading (at maximum load); steps 3–6: cyclic loading)

Figure 8 shows the average relative position of the fractured fragments in anterior and posterior aspects for the three fixation methods. For DHS + DS, the average relative position of the fractured fragments in the anterior aspect is very similar to that in the posterior aspect during loading. For PFLP, the average relative position of the fractured fragments in the anterior aspect is similar to that in the posterior aspect. However, for CSs, the average relative position of the fractured fragments in the anterior aspect is completely different from that in the posterior aspect. Moreover, for CSs, the difference in the average relative position of the fractured fragments between the anterior and posterior aspects increases during loading.

**Fig. 9:**
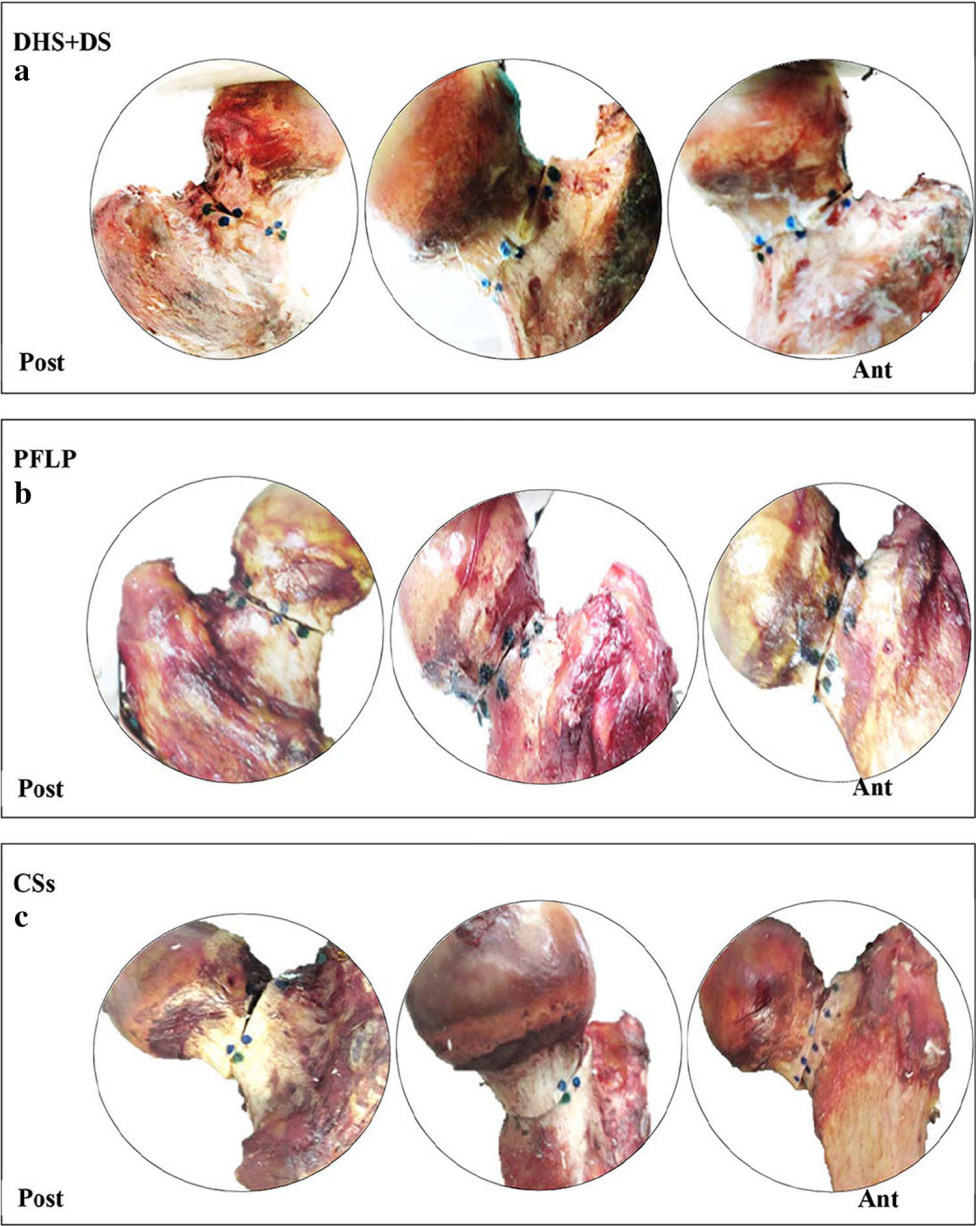
Anterior (*right*) and posterior (*left*) views of cadaveric bone samples fixed using **a** DHS + DS, **b** PFLP, and **c** CSs after 10,000 loading cycles

Figure 9 shows cadaveric bone samples fixed using DHS + DS, CSs, and PFLP at the end of 10,000 loading cycles.

### Finite Element Analysis

**Fig. 10:**
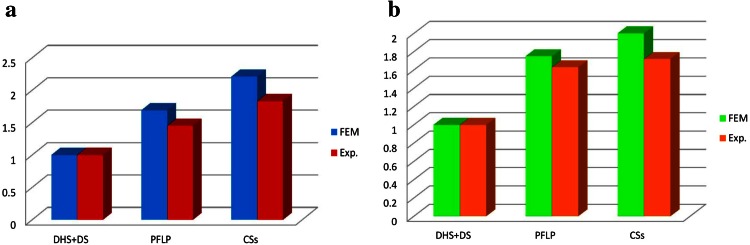
Comparison of experimental and FEA results under static loading condition for three fixation methods. **a** Normalized axial femoral head displacement and **b** normalized interfragmentary movement

Figure 10 shows the normalized axial femoral head displacement and normalized interfragmentary motion obtained in experimental tests and FEA. Under static loading, according to FEA, the normalized femoral head displacements in the vertical direction for the DHS + DS, PFLP, and CSs models are 1, 1.69, and 2.21, respectively. Moreover, the normalized values of average interfragmentary movement in the DHS + DS, PFLP, and CSs models are 1, 1.75, and 2, respectively.

Both experimental data and FE results indicate that during static loading, the DHS + DS method of fixation has the lowest axial femoral head displacement as well as the lowest interfragmentary movement.

## Discussion

Vertical femoral neck fractures, i.e., unstable Pauwels’ III fracture in which fracture orientation is greater than 70°, may experience high shear forces, and thus may be predisposed to nonunion or loss of fixation [1, 3]. Since there is controversy regarding the ideal fixation method for this kind of fracture [3], a series of experimental and computational models were developed in this study to compare the common fixation techniques used for unstable Pauwels’ III fracture. To the best of our knowledge, this is the first study that employed motion capture analysis to compare the biomechanical stability of internal implants for this kind of fracture.

The dominance of shear forces in vertical femoral neck fractures causes femoral head toggling. Hence, a stable fixation method should resist toggling during the bone healing process [3]. According to the average relative position of the fracture fragments and its components, i.e., axial and shear relative position versus load curves, the specimen fixed with the DHS + DS method provided the greatest resistance against femoral head toggling and rotation (see Figs. 5, 6, 7, 8). Thus, the DHS + DS method of fixation can keep the proximal and distal segments together more firmly (compared to PFLP and CSs) during the course of the healing process. As shown in Figs. 6, 7, the CSs and PFLP methods allow toggling, sliding, and rotating of the femoral head. It should be noted that according to the interfragmentary motion results, PFLP shows superior toggling, sliding, and rotation resistance of the femoral head compared to that for CSs (see Fig. 5, 6, 7, 8). In addition, the stiffness, axial femoral head displacement, failure load, and failure energy for the DHS + DS method of fixation prove that this method provides the strongest structure. Of note, there was no considerable difference in rigidity between the PFLP and CSs methods (see Table 1). Figure 9 shows cadaveric bone samples fixed using DHS + DS, CSs, and PFLP at the end of 10,000 loading cycles. This figure indicates that the femoral head fixed using DHS + DS had the greatest resistance against shear and rotational forces, followed by PFLP and then CSs. Considering that the union of this kind of fracture occurs during the primary bone healing process, which necessitates absolute stability at the fracture site, DHS + DS may require shorter healing time than that for PFLP, which needs less time for healing than CSs.

Both the experimental and numerical investigations in this study indicate that during static loading, the DHS + DS method of fixation allows the lowest axial femoral head displacement and interfragmentary movement, followed by PFLP and then CSs (see Fig. 10). It seems that in the static loading condition, the DHS + DS fixation method firmly clamps the fractured fragments together. Due to the similarity between the FEA results and experimental data, the FE models have great potential to predict the mechanical performance of bone-implant constructs. The differences between experimental and FEA results in this study could have been due to several simplifications made in the FE models, such as the rough pattern of cortical and spongy bone distributions, non-realistic boundary conditions, and disregard of the friction between bone and screw. For instance, there are likely some relative motions at the screw-bone interface, which were not included in this study.

In a recent study by Aminian et al. [12], the stiffness and failure load of the CSs method were reported to be 166 ± 50 N/mm and 0.862 ± 0.366 kN, respectively. Also, the stiffness and failure load of the DHS + DS technique were reported to be 277.9 N/mm and 2.32 kN, respectively, in Nowotarski et al.’s study [14]. In Nabhani et al.’s study, interfragmentary movement calculated using their FE model of femoral neck fracture at an angle of 60° with respect to the horizontal axis, which was fixed with three CSs in an inverted triangle arrangement, was reported to be 0.05 mm [34]. In the present study, the interfragmentary movement derived using our FE model for the femur fixed by CSs, was 0.034 mm. The differences between the present study’s results and those of previous studies might be due to the differences in loading regimes, assumptions made regarding loading and boundary conditions, load application device, femur orientations, fracture orientation, femur type, and implants used in the respective studies. Similar to previous studies [11–14, 19], the results of this research show that fixed-angle devices are stronger than CSs for the fixation of vertical femoral neck fracture. In recent studies by Aminian et al. [12] and Nowotarski et al. [14], femoral neck locking plates were reported as the strongest fixation method for vertical femoral neck fracture. It should be noted that the locking plates used in these studies [12, 14] were different from the PFLP employed in this research. In this study, PFLP with two locking screws was compared with DHS + DS and CSs. However, in Aminian et al.’s study [12], the Synthes PFLP (Synthes, Paoli, PA, USA) with three locking screws was compared to DHS, DCS (dynamic condylar screw), and CSs, and in Nowotarski et al.’s 2012 [14], a newly designed PFLP with two locking screws and a transfer lag screw was compared with DHS + DS and CSs. Based on our results, because PFLP with its two locking screws could not compress the fracture fragments adequately, it was less stable than DHS + DS, which provided a stronger support at the inferior location around the fracture site.

Similar to other studies, there were several limitations in this research. First, few experimental models were developed in this study. Second, the osteotomy was created using a smooth saw cut, which is different from a real bone fracture surface. Third, interfragmentary movements were evaluated in a 2D space, whereas interfragmentary motions occur in three dimensions, and thus are better captured in a 3D space. Fourth, to reduce the computational complexity in the FE analysis, bone-screw coupling was assumed to be a tie contact, although in order to simulate a real situation, this coupling should be modeled using a frictional contact model. Fifth, for better evaluation of fixation methods using FE analysis, cyclic loading should also be applied to the models in addition to the static loads. Finally, for simplicity, physiologic force components acting across the hip joint, such as muscle forces, were neglected in this research.

The novelty of this study was the use of motion capture analysis as an experimental tool to compare three common fixation methods for vertical femoral neck fracture. By applying this tool, negative aspects of fixation techniques such as toggling and shear displacements, which are signs of instability and failure of fracture union, can be investigated. Moreover, in this study, FEA was used with the primary goal of comparing various fixation methods by measuring the interfragmentary motions, with the ultimate goal of assessing more important parameters, such as the stress or strain distribution within the bone and implant, which are difficult and likely impossible to experimentally measure. In the future, in order to investigate the effects of various engineering designs of screws on the stress shielding in the bone-implant construct, a more realistic assumption for the bone-screw interface [35], as well as the employment of bone remodeling theories [36], will improve our understanding of this problematic fracture in young patients.

## Conclusion

The aim of this study was to compare the biomechanical performance of three fixation methods, namely CSs, DHS + DS, and PFLP, for femoral neck fractures. The results of this research suggest that, based on the clinical assumption that restricted weight-bearing regimen is recommended in the postoperative rehabilitation protocol, the DHS + DS method of fixation is more effective compared to CSs and PFLP for vertical femoral neck fracture fixation in young adults, and may reduce healing time.
